# Committee Proceedings on the Bill

**Published:** 1919-04-12

**Authors:** 


Provisional Nursing Council.

### COMMITTEE PROCEEDINGS ON THE BILL.

The consideration of the Bill in Committee was begun on Tuesday, Mr. D. Macmaster, K.C., presiding, and Clauses 1, 2, and 3 were agreed to without amendment.

On Clause 4, which fixes the constitution of the Council, an amendment, proposed by Sir Edgar Jones, that the number be raised from 41 to 42 to allow for the representation of Wales, was agreed to.—Another amendment, moved by Mr. Briant, that the Council consist of 45, was also agreed to. Mr. Briant's object is to ensure that three persons not connected with the medical or nursing professions shall be on the Council.

Lieut.-Col. Raw, in moving the first of his amendments (set out above), said his object was to secure a majority of nurses on the Council which was to regulate their profession.—Dr. Addison (President of the Local Government Board) said the Bill as it stood allowed for 24 members of the nursing profession out of 45.—The amendment was ruled out of order on the ground that it would involve "fractions of nurses." (Laughter.)

Lieut.-Col. Raw then moved his second amendment (given above) to Clause 4.—Major Barnett (one of the promoters of the measure) opposed. He pointed out that the College of Nursing claimed that they had 13,000 on their Register. With the addition of another 7,000 they would have a controlling voice in the future of the nursing profession.—Dr. Addison suggested that the question of the constitution of the Council should be considered by the Government Departments concerned and the Medical and Nursing Associations. The amendment before them seemed to take no cognisance of the State, which had a responsibility of a very heavy character.—Mr. John Hodge objected to the Privy Council nominating members. A democratic selection was wanted, and it should be made by the Ministry of Health.—Lieut.-Col. Raw withdrew his amendment, and the Committee adjourned till April 10, by which time it is hoped to come to some agreement as to the constitution of the Provisional Nursing Council.

